# Mortality and failure among tuberculosis patients who did not complete treatment in Vietnam: a cohort study

**DOI:** 10.1186/1471-2458-7-134

**Published:** 2007-07-02

**Authors:** Marleen Vree, Nguyen T Huong, Bui D Duong, Dinh N Sy, Le N Van, Nguyen V Co, Frank GJ Cobelens, Martien W Borgdorff

**Affiliations:** 1 KNCV Tuberculosis Foundation, The Hague, The Netherlands; 2 Center for Infection and Immunity Amsterdam (CINIMA), Academic Medical Center, Amsterdam, The Netherlands; 3 National Tuberculosis Programme Vietnam, Hanoi, Vietnam; 4 National Reference Laboratory, National Hospital for Tuberculosis and Respiratory Diseases, Hanoi, Vietnam

## Abstract

**Background:**

Tuberculosis treatment failure and death rates are low in the Western Pacific Region, including Vietnam. However, failure or death may also occur among patients who did not complete treatment, i.e. reported as default or transfer-out. We aimed to assess the proportion failures and deaths among new smear-positive pulmonary tuberculosis patients with reported default or transfer-out.

Treatment outcomes rates were 1.4% default, 3.0% transfer-out, 0.4% failure and 2.6% death in northern Vietnam in 2003.

**Methods:**

Tuberculosis patients in 32 randomly selected district tuberculosis units in northern Vietnam were followed up 1 to 3 years after treatment initiation for survival, recent treatment history and bacteriologically confirmed tuberculosis.

**Results:**

Included were 85 transferred patients and 42 who defaulted. No information was available of 41 (32%), 28 (22%) had died. Fifty-eight were available for follow-up (46%); all had sputum smear results. Tuberculosis was recorded in 11 (13%), including 6 (7%) with positive sputum smears, 3 (3%) with negative smears but positive culture and 2 (2%) who had started re-treatment for bacteriologically confirmed tuberculosis. Fifteen (17%, 95%CI 10–27%) had died within 8 months after treatment initiation. Of 86 patients with known study outcomes, 39 (45%, 95%CI 35–56%) had died or had bacteriologically confirmed tuberculosis. This was recorded for 29/53 (55%, 95%CI 40–68%) transferred patients and 10/33 (30%, 95%CI 16–49%) patients who defaulted.

**Conclusion:**

The total failure and death rates are 0.6% and 0.8% higher than based on routine reporting in northern Vietnam. Although this was a large proportion of treatment failures and deaths, failure and death rates were low. Defaulting and transfer carry a high risk of failure and in particular death.

## Background

The global strategy for tuberculosis of the World Health Organization (WHO), the DOTS strategy, recommends the use of short-course chemotherapy (SCC). Tuberculosis treatment outcomes are reported according to internationally accepted definitions [1]. These include cure, treatment completion, treatment failure, death, default and transfer-out. Specific criteria are defined for cure, treatment failure and death. However, patients who did not complete treatment at the district tuberculosis unit, i.e. reported as default or transfer-out, may include undetected deaths and treatment failures. The consequence would be that the overall treatment effectiveness, i.e. the proportion of patients without death or treatment failure, would be lower than can be assessed from the routine reporting of treatment outcomes.

A defaulter is defined as a patient whose treatment was interrupted for 2 months or more. A transfer-out is defined as a patient who has been transferred to another recording and reporting unit and for whom the treatment outcome is not known [1].

Default and transfer-out rates are low with 2.0% and 1.7% in the Western Pacific Region [2], but correspond to over 15,700 cases in 2003.

High morbidity and mortality rates are reported after treatment default, but estimates differ. Mortality after default was 4% in Singapore [3] and 27% in Mexico [4]. Failure after default was 28% in South Africa [5] and 54% in the USA [6]. All studies, except the study from Mexico [4] were carried out in urban settings and follow-up time differed greatly between studies. Furthermore, as these are competing risks, the cumulative burden of morbidity and mortality is unknown. Moreover, no studies have reported on the bacteriologic and survival status after transfer-out.

Vietnam, one of the 22 high-burden countries for tuberculosis [7] report high treatment success rates (92%) of new smear-positive tuberculosis patients who started treatment in 2002 [7], but with a high relapse and death rate 1 to 2 years after treatment completion [8]. Reported rates of treatment failure (0.8%) and death (3.4%) were low [7]. This reported failure rate based on a single smear examination in routine practice is very low compared to the failure rate of 5% based on culture at treatment completion in a clinical controlled trial with an 8-month treatment regimen as is used in Vietnam [9].

We hypothesized that treatment failure and death rates are higher if these patients are hidden in reported default or transfer-out. In order to quantify this, we assessed the proportion treatment failures and deaths based on survival, the history of re-treatment and sputum smear examination and culture after 1 to 3 years after treatment initiation of new smear-positive pulmonary tuberculosis patients with reported treatment default or transfer-out in northern Vietnam.

Characteristics of the National Tuberculosis Control Programme (NTP) Vietnam are described elsewhere [10]. Treatment outcomes rates were 1.4% for default, 3.0% for transfer-out, 0.4% for failure and 2.6% for death in northern Vietnam in 2003 [11]. In northern Vietnam in 1996 the prevalence among new smear-positive patients were 17%, 19% and 1.1% for isoniazid, streptomycin and multidrug resistance, respectively [12]. The HIV prevalence was estimated at 0.5% in the adult population in 2005 [13]. The standard NTP treatment regimen for new patients consists of streptomycin, isoniazid, rifampicin and pyrazinamide for 2 months, followed by isoniazid and ethambutol for 6 months, all with daily drug intake (2SRHZ/6HE).

## Methods

The study was conducted in 32 randomly selected NTP tuberculosis units in 22 provinces in northern Vietnam.

Eligible for inclusion in the cohort in the selected districts were all patients diagnosed with previously untreated smear-positive tuberculosis and with a reported treatment outcome of default or transfer-out and who had started treatment between 1 January 2002 and 31 December 2003. Excluded were patients ≤15 years and patients living outside the district at the time of treatment.

Patient data were recorded on a pre-coded structured questionnaire. Data on diagnosis, treatment and re-treatment were extracted from routine registers at the district tuberculosis unit.

The Research board of the National Hospital for Tuberculosis and Respiratory Diseases in Hanoi gave scientific and ethical clearance for this study. Patients who were identified as smear- or culture-positive at follow-up were offered tuberculosis re-treatment.

Trained district tuberculosis staff collected the data and provincial staff and one of the authors (MV) supervised the study. In November and December 2004 the selected patients were invited to the district unit. After written informed consent two sputum samples were collected on the spot; one before and one after the interview. Patients who did not report were visited at their homes. If patients could not be found or had died a non-response interview was completed with a family member.

Sputum smear microscopy and mycobacterial culture were performed at two provincial tuberculosis laboratories and the National Tuberculosis Reference Laboratory in Hanoi (NTRL). Sputum specimens were transferred to the laboratory within 2 days after collection and examined using the Ziehl-Neelsen method. For each patient a single mycobacterial culture was performed on Ogawa medium using the simple decontamination method (i.e. without centrifugation) [14]. *M. tuberculosis *was identified by the niacin test. Drug susceptibility testing (DST) was done at the NTRL by the proportion method [15]. All negative slides of patients who had positive cultures and positive smear slides of patients who had negative cultures were reread by technicians blinded to the original results. All smear slides read at one and 20% of slides read at another provincial laboratory were reread at the NTRL.

A case was defined as smear-positive if at least one sputum smear examinations was positive. A failure case was defined as a patient who was smear or culture-positive at the time of follow-up or had been diagnosed with smear-positive tuberculosis and started re-treatment at the district tuberculosis unit in the interval between start of initial treatment and time of follow-up.

### Data analysis

Data were double-entered using Epi Info 2002. Inconsistencies were checked against raw data. Analyses were performed using SPSS 11.5 (SPSS Inc, USA) and Stata/SE V8.0 (Stata Corp., College Station TX, USA).

The proportion undetected failures was calculated as the proportion bacteriologically confirmed tuberculosis among patients with default or transfer-out times the proportion of patients with default (1.4%) or transfer-out (3.0%) in northern Vietnam in 2003 [11]. Death during tuberculosis treatment is internationally defined as a patient who dies for any reason during the course of treatment [1] which is 8 months in Vietnam. Therefore, we analyzed the proportion of deaths within 8 months after treatment initiation. Thus, death rates in this study could be compared to death rates as reported by the NTP. The proportion undetected deaths within 8 months after treatment initiation was calculated as the proportion death among patients with treatment default or transferred out times the proportion of patients with treatment default (1.4%) or transfer-out (3.0%) patients in northern Vietnam in 2003 [11].

To assess differences at the 5% significance level two-sided Fisher's exact test and Chi^2 ^test with likelihood ratios were used. Survival curves up were constructed using the Kaplan-Meier method.

## Results

Included in the cohort were 42 patients with treatment default and 85 patients with transfer-out. Time of default or transfer-out was within the first 2 months of treatment (initial phase) for 10 (24%) of all patients with default and for 47 (57%) of all patients with transfer-out. Follow-up sputum smear result at 2 months after treatment initiation was known for 25 patients with default and 25 patients with transfer-out. Of these, 0 (0%) patients with default and 2 (8%) patients with transfer-out were sputum smear-positive.

**Figure 1:**
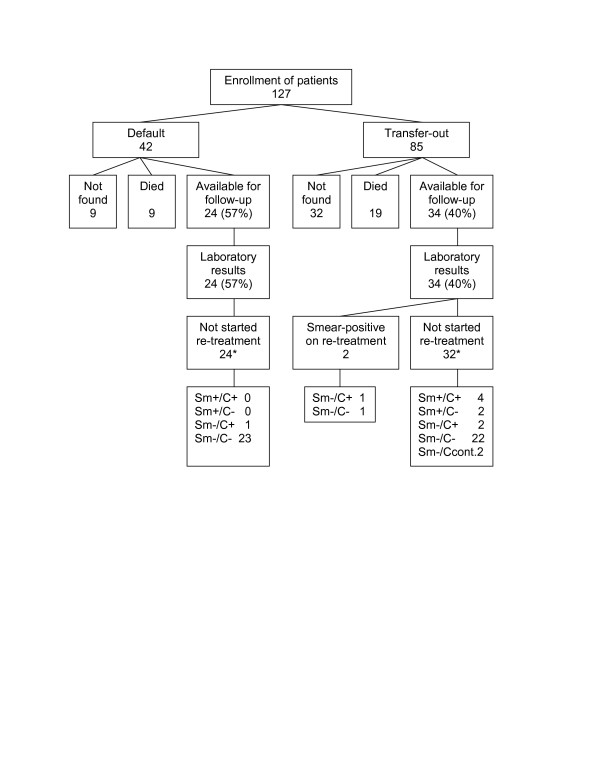
Study outcomes at time of follow-up of tuberculosis patients with treatment default or transfer-out. sm = smear. C = culture. Ccont = contaminated culture. * = according to the laboratory and treatment register of the district where the patient was initially treated.

No information was available of 41 (32%) patients (Figure 1); of these 17 (35%) had moved and 6 (13%) were released from prison (Table 1). The time interval between treatment initiation and the date the patient was last seen at the district tuberculosis unit was available for 24 patients with non-response (59%). The median interval was 5 months (inter-quartile range (IQR) 2 to 21 months).

**Table 1:** Characteristics of non-responders at time of follow-up of tuberculosis patients with treatment default or transfer-out.

	Default		Transfer out	
		
	Non-response n (% of all in subgroup)	p†	Non-response n (%)	p†
All	9 (21%)		32 (38%)	
Age, years		0.20		0.42
15–34	6 (35%)		9 (50%)	
35–54	1 (13%)		12 (38%)	
≥55	2 (12%)		11 (31%)	
Sex		0.46		0.49
Men	6 (27%)		22 (41%)	
Women	3 (15%)		10 (32%)	
Area		0.81		0.61
Urban	3 (20%)		9 (45%)	
Mountainous/remote	2 (17%)		7 (30%)	
Rural	4 (27%)		16 (38%)	
Reason for non-response				
Moved	6		11	
Released from prison	0		6*	
Never lived here	0		1	
Other	1		0	
Unknown	2		14	

* of whom 2 with HIV-infection

† P-values based on difference between patients with non-response and patients who had died or were available for follow-up.

Twenty-eight patients had died (22%). Fifty-eight patients were available for follow-up (46%) and all had interview data and smear results (Figure 1). The median time interval between treatment initiation and follow-up was 22 months (IQR 16 to 29 months).

Interview data showed that of patients with transfer-out one had been transferred to the commune health center, one to the district health center, 15 (44%) to the provincial tuberculosis treatment center and nine to a national tuberculosis referral hospital. One had not been transferred and for 7 this remained unknown.

At the time of follow-up 10 of 86 patients with known study outcomes (11%, 95%CI 5.7–20%) had positive sputum smears or cultures (Figure 1). Six patients who had not been re-treated by time of follow-up were smear-positive and an additional three were culture-positive (Figure 1). Two patients had started re-treatment for smear-positive tuberculosis between interrupting initial treatment and follow-up; one of these patients was culture-positive at time of follow-up. In addition, two patients with default stated in the interview to have received re-treatment, but sputum smear status was unknown. Therefore, 11 (13%, 95%CI 6.6–22%) patients in total had bacteriologically confirmed tuberculosis during time of follow-up. Of these, all but one patient were transfer-out and the proportion failure among them did not differ significantly by age group, sex or area (Table 2).

**Table 2:** Failure and death at time of follow-up of tuberculosis patients with treatment default or transfer-out.

	*Default*			Transfer-out						Total		
				
	*Death*	Total*		Failure	Total^†^		*Death*	Total*		Death = 8 months after treatment initiation	Total*	
	*n (%)*	*n*	*p*	n (%)	n	p	*n (%)*	n	p	n	n	p
All	9 (27%, 95%CI 13–46%)	33		10 (29%, 95%CI 15–47%)	34		19 (36%, 95%CI 23–50%)	53		15 (17%, 95%CI 10–27%)	86	
Age, years			0.002			0.85			0.74			0.100
15–34	0 (0%)	11		2 (33%)	5		4 (44%)	9		1 (5%)	20	
35–54	1 (14%)	7		4 (29%)	14		6 (30%)	20		4 (15%)	27	
≥55	8 (53%)	15		4 (27%)	15		9 (37%)	24		10 (26%)	39	
Sex			0.78			1.0			1.0			1.00
Men	4 (25%)	16		6 (29%)	21		11 (34%)	32		8 (17%)	48	
Women	5 (29%)	17		4 (31%)	13		8 (38%)	21		7 (18%)	38	
Area			0.019			0.47			0.32			0.074
Urban	1 (8%)	12		3 (43%)	7		4 (36%)	11		1 (4%)	23	
Mountainous/remote	6 (60%)	10		3 (37%)	8		8 (50%)	16		7 (27%)	26	
Rural	2 (18%)	11		4 (21%)	19		7 (27%)	26		7 (19%)	37	

* total = number of deaths & number with known survival

^† ^total = number of patients who did not have bacteriologically confirmed tuberculosis

Drug resistance results could be obtained for seven of eight positive cultures. Of these, three were multidrug resistant (Table 3).

**Table 3:** Tuberculosis drug susceptibility of patients with treatment default (n = 1) or transfer-out (n = 7) and with positive *M. tuberculosis *cultures at follow-up.

	Default n	Transfer-out n
Fully susceptible	1	1
Resistant to		
H	0	2
HRS	0	1
HRES	0	2^†^
No information	0	1

S = Streptomycin, H = Isoniazid, R = Rifampicin, E = Ethambutol

^† ^including 1 patient on re-treatment regimen

**Figure 2:**
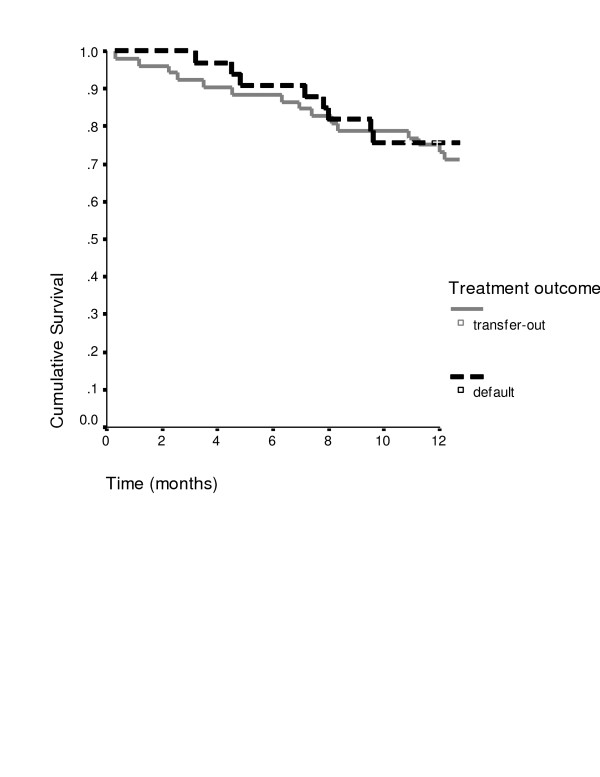
Survival of tuberculosis patients with treatment default or transfer-out during 1 year after treatment initiation.

Death was recorded for 28 of 86 patients with known study outcomes (33%, 95%CI 23–44%). Among 27 deceased patients the median time interval from treatment initiation to death was 8 months (IQR 5 to 11 months); for 1 patient this remained unknown. Fifteen (17% of all patients with known study outcomes, 95%CI 10–27%) patients had died within 8 months after treatment initiation (Table 2 and Figure 2). The proportion death within 8 months after treatment initiation did not differ significantly by age group, sex or area (Table 2). Reported causes of death were tuberculosis for 11, lung disease for 6 patients, HIV/AIDS for 3, other for 4 and unknown for 4. Mortality increased with age among patients with default, but did not differ significantly for patients with transfer-out (Table 2).

Death or bacteriologically confirmed tuberculosis was recorded for 39 patients (45%, 95%CI 35–56%) during the total follow-up time. This was recorded for 29/53 (55%, 95%CI 40–68%) patients with transfer-out and 10/33 (30%, 95%CI 16–49%) patients with default.

**Figure 3:**
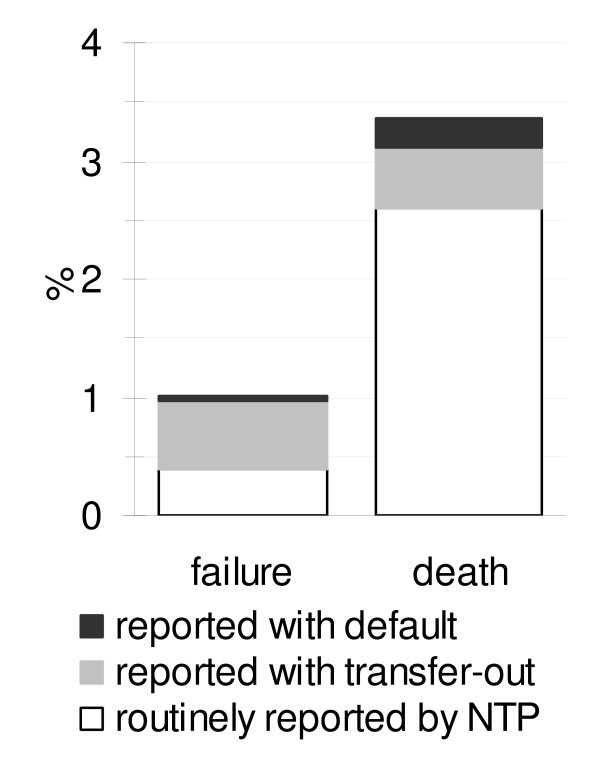
Proportion failures and deaths during treatment duration among tuberculosis patients with treatment default or transfer-out.

Undetected treatment failures were observed among 0.6% of patients who started treatment and hidden in default or transfer-out. This is 60% of all treatment failures, i.e. of treatment failures among patients with default or transfer-out plus the treatment failures routinely reported by the NTP in northern Vietnam (0.4%) (Figure 3). Undetected death within 8 months after treatment initiation were observed among 0.8% of patients who started treatment and hidden in default or transfer-out. This is 24% of all deaths, i.e. of deaths among patients with default or transfer-out plus the deaths routinely reported by the NTP in northern Vietnam (2.6%) (Figure 3).

## Discussion

Of new-smear positive tuberculosis patients with default or transferred to other tuberculosis units, 45% had died or had bacteriologically confirmed tuberculosis after 1–3 years follow-up in northern Vietnam in 2003. Sixty percent of treatment failures and 24% of deaths within 8 months after treatment initiation remained undetected and therefore were unreported in the routine reporting system. Consequently, the total failure and death rate were 1.0% and 3.4%.

Eleven patients (13%) had sustained or developed bacteriologically confirmed tuberculosis during the follow-up period. This is similar to the 10% combined failure and relapse rate observed in a randomized-controlled trial without rifampicin in the continuation phase [9]. However, this 13% was lower than in South Africa where 28% of defaulters had recurrent tuberculosis as diagnosed through passive case finding [5] and lower than in the USA where 54% patients with default had a positive culture within 1 month of the last recorded dose [6]. Possible explanations for this relatively low failure rate in Vietnam may be longer treatment duration with better treatment compliance before default, or a higher proportion of patients with tuberculosis had died.

With 33%, mortality after treatment default or transfer-out was high. This was similar to 27% deaths due to tuberculosis in Mexico [4] during a 2.3 year follow-up period after diagnosis. However, the high mortality in our study may only in part be attributable to tuberculosis, since the reported cause of death was tuberculosis for only 11 (of 28) patients. However, the cause of death as reported by family members is probably not accurate and death attributed to tuberculosis can be under- or overestimated. Another explanation for the high mortality observed is the emerging HIV epidemic in Vietnam [16]. Vietnam has no routine system of HIV testing of tuberculosis patients, and the HIV-status of the patients in our study was largely unknown. In the current situation in Vietnam, HIV infected patients are likely to be transferred to provincial or national hospitals, since the district tuberculosis unit may not be able to provide adequate health care. HIV prevalence may therefore be higher among patients reported as transfer-out compared to patients with reported treatment success. Furthermore, it is unknown how many deaths could have been prevented if treatment was continued in time. In this study 24 of 42 patients who defaulted were available for a follow-up health examination. Therefore, active follow-up of patients who did not complete treatment may be an effective intervention to ensure treatment completion and thereby lower the high morbidity and mortality.

A study limitation is the large proportion of patients for whom the status at follow-up remained unknown. If all these patients were alive at the time of follow-up and either none or all had bacteriologically confirmed tuberculosis, the failure rate would be either 9% or 41%. Therefore, the total failure rate is estimated to be 0.4% to 1.8% higher than based on routine reporting. If all non-responders were either alive or had died at time of follow-up, the death rate would be either 22% or 54%. Therefore the total death rate is estimated to be 0.5% to 1.3% higher than based on routine reporting. These lower and upper estimates confirm the finding that a large proportion of failure and deaths remain undetected among patients with default or transfer-out.

Another study limitation is the use of the simple method for culture [17], which may have resulted in 5–10% negative cultures, especially when bacillary load is low. Consequently, one or two additional culture positive cases are expected, but that would increase the number of failure cases only slightly. In contrast, some bacteriologically confirmed tuberculosis cases may in fact be due to reinfection rather than treatment failure [18]. Reinfection occurred among 11% of defaulters in South Africa, but here the estimated tuberculosis incidence is substantially higher than in Vietnam [5]. Therefore we conclude that around 13% of patients with default or transfer-out were treatment failures.

A large proportion of treatment failures remain currently undetected by the NTP and those patients are not detected for further tuberculosis treatment. In this study only two of eleven patients had started re-treatment. In northern Vietnam in 2003 this would be 105 undetected failure cases as compared to 48 cases who received re-treatment after failure. Moreover, in this study 3 of 7 (43%) patients carried multidrug resistant strains of those with culture-positive tuberculosis. Patients with primary multidrug resistance have a high risk of death and failure with the standard NTP treatment regimen [19]. These patients may not recover during treatment and may be transferred for more adequate health care or a place to die. Therefore, multidrug resistance may explain the high combined failure and death rate of 55% among patients with transfer-out. As routinely reported failure cases, these undetected failure cases are likely to contribute to continued transmission of drug resistant tuberculosis.

Failure and death rates among patients with transfer-out may reflect that of patients with default or with completed treatment. This depends on whether patients with transfer-out continued and completed treatment in another NTP treatment center. This information will guide the NTP how to lower the high risk of death and failure. Therefore, it is recommended to evaluate the actual treatment outcome of patients registered with transfer-out at the provincial and national level. Forty-four percent of patients reported to be transferred from the district tuberculosis unit to the provincial hospital. As a consequence, a specific treatment outcome, i.e. cure, treatment completion, death or failure, can be given for around half of the number of patients with transfer-out.

These findings may be important beyond Vietnam to countries with a similar tuberculosis epidemiology and reported treatment outcomes, like many countries in Southeast Asia [2]. A follow-up study among patients who did not complete treatment may be considered in settings with high default or transfer-out rates, such as in Eastern Europe, or among patients who started treatment in institution other than ambulatory settings, such as hospitals or prisons.

## Conclusion

Tuberculosis patients with treatment default or transfer-out carry a high risk of failure and in particular death. Of tuberculosis patients with treatment default or transfer-out 13% were actually treatment failures and 17% were actual deaths within 8 months after treatment initiation in northern Vietnam. Therefore, in this setting the total treatment failure and death rates are 60% and 24% higher than based on routine reporting (0.4% and 2.6%). Although treatment failure and death rates are low, a large proportion of treatment failures and deaths remain undetected. This is of importance, since undetected failure cases are likely to contribute to continued transmission of drug resistant tuberculosis.

## Competing interests

The author(s) declare that they have no competing interests.

## Authors' contributions

MV, FC, MB are responsible for the study design. MV, NH, BD, DS, LV and NC contributed to the acquisition of the data. MV planned and conducted the study and drafted the first version of the manuscript. All authors have been involved in revising it critically for important intellectual content. All authors read and approved the final manuscript.

## Pre-publication history

The pre-publication history for this paper can be accessed here:


